# COVID-19: the crucial role of the nose

**DOI:** 10.1016/j.bjorl.2020.08.001

**Published:** 2020-09-16

**Authors:** Mario Gamerra, Eugenio de Corso, Elena Cantone

**Affiliations:** a ASL Distretto 56 Napoli 3 Sud, Torre Annunziata, Italy; b Policlinico Universitario A. Gemelli/Università Cattolica del Sacro Cuore, Otorinolaringoiatria, Rome, Italy; c Federico II University of Naples, Department of Neuroscience, Reproductive and Odontostomatological Sciences, Ear Nose Throat Section, Naples, Italy

Dear Editor,

We have followed with a great interest the article by Lavinsky et al. and herein we present a simple additional therapeutic strategy against SARS-COV-2.^1^

As recently demonstrated, climatic factors such as latitude, temperature, and humidity strongly influence the propagation of the COVID-19 virus.^2^ According to Sterling, some viruses prefer a high relative humidity, whereas some other viruses a low relative humidity, so there is an average humidity range between 50% and 70% in which the viral population is minimal.^3^ However, current climatic transformations are determining strong thermal excursions, which could destabilize the climate and favor the survival of the virus. Although it is not realistically possible to change the global climate in the short term, changing indoor humidity and temperature in workplaces, schools, hospitals especially intensive care units could be a valid preventive and therapeutic strategy to reduce respiratory infections.^3^

The nose represents the first contact with inhaled pathogens and the target organ for SARS-CoV-2, due to the presence of virus receptors in the rhinopharynx. The anterior nasal segment and turbinates regulate temperature and humidity of inhaled air to near alveolar condition to protect the lungs. Although unlikely, drying of the mucous membranes of the nose and throat might increase susceptibility to respiratory infections.^4^ However, these phenomena might explain the higher vulnerability of elderly subjects to the viruses, who often complain of nasal dryness due to the degenerative effects of aging on nasal mucosa. Conversely, mucus might somehow, as in the pediatric population, prevent respiratory infections by preventing contaminated aerosols from reaching the lungs. These speculations do highlight the importance of preserving a good nasal physiology in the preparation of the inhaled air before reaching the lungs; cigarette smoking, intranasal vasoconstrictors or steroids, and previous turbinectomy operations, reducing nose moisture, could be predisposing factors to viral infection. An additional therapeutic strategy against SARS-COV-2 could be the creation, in both upper and lower airways, of conditions of temperature and humidity not favorable to the virus. In particular, humidification of the lungs might be useful in patients hospitalized for pneumonia, especially when oxygen therapy is needed.^5^ For these reasons, the elderly, much more than young people, could benefit from a humidifying therapy of the intranasal air.

## Conflicts of interest

The authors declare no conflicts of interest.
